# A Second Species of *Etiennea* (Coccidae: Coccoidea: Sternorrhyncha) from the New World

**DOI:** 10.1673/031.007.5101

**Published:** 2007-10-11

**Authors:** Chris Hodgson, Takumasa Kondo

**Affiliations:** ^1^ Department of Biodiversity and Biological Systematics, The National Museum of Wales, Cardiff, CF10 3NP, Wales.; ^2^ Department of Entomology, University of California, 1 Shields Avenue, Davis, California 65616, USA

**Keywords:** Burseraceae, soft scale insect

## Abstract

The genus *Etiennea* Matile-Ferrero (Coccidae: Coccoidea) currently contains 19 species, all but one of them restricted to Africa, the exception being from Guyana. The present paper describes the adult female of a further species, *Etiennea bursera* sp. nov., from the New World (Mexico). The key in Hodgson (1991) is augmented to separate the new species from the others in the genus. The relationships of *Etiennea* to other coccid genera are briefly discussed.

## Introduction

The genus *Etiennea* was erected by Matile-Ferrero (1984) to take a rather distinct soft scale species, *E. villiersi* Matile-Ferrero from Senegal. The genus *Etiennea*, along with *Platysaissetia* Cockerell, was revised by Hodgson (1991) who added 12 new species and transferred 6 further species to it, mainly from *Platysaissetia*. All but one of these species were known only from Africa, the sole exception being *E. montrichardiae* (Newstead) from Guyana. The present paper describes a second species from the New World off *Bursera* sp. (Burseraceae), imported into the USA from Mexico. As the genus *Bursera* is restricted to tropical America (Willis 1973), it seems likely that this new species originated from there, thus constituting the second record of this genus in the New World. Only two non-cosmopolitan genera of Coccidae are currently known from both Africa and South or Central America, *Alecanochiton* Hempel (including *Avricus* De Lotto, see Hodgson 1994) and *Etiennea*, thus the discovery of a further New World species in the latter genus is significant. Species in two other non-cosmopolitan, mainly Neotropical, genera (*Akermes* Cockerell and *Toumeyella* Cockerell) have been recorded from both Africa and the New World in the past, but it is now believed that the African species are not congeneric with the type species in either genus (Kondo 2006; Kondo 2007).

## *ETIENNEA* Matile-Fererro 1984

*Etiennea* Matile-Fererro 1984:100.

### *Etiennea bursera* Hodgson & Kondo sp. nov.

(Figure 1)

### Material examined

**HOLOTYPE : MEXICO**, intercepted in USA, on *Bursera* sp., 3.ii.2005, B. Abijoy (USNM): 1/1 adult 9, young and in good condition.

**PARATYPES:** data as for holotype (USNM): 4/4 adult ♀♀ , in good condition but 1 partially sclerotised and other 3 heavily sclerotised.

Described mainly from holotype specimen, with some details checked on 2 other specimens, these indicated below by ranges of data.

### Unmounted material

Not seen.

### Mounted material

Length 1.9–2.5 mm, width 1.5–2.5 mm. Body oval, longer than wide when young, becoming wider than long when old. Derm thick, with a small area of areolated sclerotisation around anal plates and dorsal to mouthparts on young specimens, becoming densely sclerotised throughout on old material, with abundant, dense, cell-like areolations, each with a paler pore; anal cleft closely adpressed, about 1/4–1/6th body length. Marginal spines marginal on young specimens, but appearing ventral on older specimens when a pseudomargin developes; stigmatic clefts shallow, each with 3 stigmatic spines. Antennae and legs well developed.

### Dorsum

Derm of young specimens membranous, with pale oval areolations, each areolation rather variable in shape but perhaps radiating rather approximately from an area dorsad to mouthparts and from around anal plates; each areolation with a microductule. Derm later becoming heavily sclerotised throughout as described above. Dorsal setae each spinose, those medially longer (15–18 µm) than most lateral setae (11–13 µm), each with a heavily sclerotised basal-socket; present fairly randomly throughout dorsum, including sclerotised areas; perhaps least abundant marginally. Dorsal microductules present in each dermal areolation, each round, 2.5–3.0 µm in diameter, with a long inner ductule. Simple pores each about 1 µm in diameter, most abundant submarginally but present very sparsely throughout. Dorsal tubular ducts absent. Preopercular pores each circular and roundly convex, 9–10 µm wide, with a granulate surface; present in a loose, broad group of about 30 in front of anal plates; perhaps restricted to abdomen. Dorsal tubercles each large, round, sclerotised, without satellite tubular ducts in outer sclerotised ring; each tubercle about 18–22 µm in diameter; present in a submarginal ring, with 3–5 per side, mainly on abdomen, none on thorax. Pocket-like sclerotisations present, each about 1/4th width of dorsal tubercles, rather variable in shape; situated between dorsal tubercles in submarginal ring; with 1–5 per side, mainly on abdomen, none on thorax. Anal plates together quadrate, each 170–183 µm long, combined widths 128–145 µm , each plate with outer angle rather rounded and posterior margin slightly longer than anterior margin; each plate with 4 pairs of setae: anterior inner margin setae at about mid-point along inner margin, each about 13 µm long; posterior inner margin seta larger, with a flagellate apex, about 36 µm long; apical and outer margin setae both about 8 µm long, latter near apex. Ano-genital fold with about 5 setae along anterior margin, longest perhaps 33 µm long, and probably with 2 pairs of short setae laterally. Anal ring heavily sclerotised; number of setae present uncertain, probably 8, each about 150 µm long.

### Margin

Marginal setae strongly spinose, rather similar to dorsal setae but slightly smaller, each seta 10–18 µm long, up to about 25 µm near anal cleft; anal lobe setae much longer, up to 60 µm long; each with a heavily sclerotised basal-socket; with 15–21 between eyespots, and with (on each side) 8–11 between eyespots and anterior stigmatic clefts; 16–18 between stigmatic clefts and 39–56 between stigmatic clefts and anal clefts. Stigmatic clefts shallow, each with 3 stigmatic spines, median spines each about 75–80 µm long, with a basal socket similar to those of marginal setae; lateral spines each 23–35 µm long, with narrower basal sockets. Eyespots marginal; each 27–28 µm wide.

### Venter

Derm entirely membranous. Pregenital disc-pores each mainly with 10 loculi and about 6–7 µm wide; with rather few around genital opening and then apparently absent medially on more anterior abdominal segments, but present as a sparse submedial band laterad to coxae as far forward as anterior spiracles; a few also present medially on thorax. Spiracular disc-pores each with mainly 5 loculi; present in a narrow band between each spiracle and margin, with about 32–48 in each band. Ventral microducts present throughout venter, subequal in size to dorsal microductules. Tubular ducts present, of one type, each with a fairly long outer ductule (14–17 µm long) and a slightly narrower inner ductule (10–11 µm long) with a large terminal gland; present in a fairly wide submarginal band. Ventral setae: with 4 pairs of interantennal setae, longest about 50 µm long; with pairs of long pregenital setae in abdominal segments VI–VIII; submarginal band sparse, with fine, short setae; other setae very sparse, but with a distinct concentration along anterior band of spiracular disc-pores. Spiracles normal but relatively small; width of each peritreme: anterior 40–48 µm, posterior 59–68µm. Legs normal; pro-tibia and tarsus fused on only clear specimen, with a strong indentation on dorsal margin; other tibia and tarsi with a distinct articulation but with no articulatory sclerosis; all segments with few setae; claws without a denticle; with one claw digitule broader than other; tarsal digitules subequal in length to claw digitules; dimensions of metathoracic legs (µm): coxa 132; trochanter + femur 145–178, tibia + tarsus 185–225; claw 27–28. Antennae each with 6 segments; total length 327–356 µm. Clypeolabral shield about 200 µm long.

### Comments

The adult female of *E. bursera* appears to belong to the group of *Etiennea* species in which the dorsal tubercles lack satellite tubular ducts and which lack tubular ducts elsewhere on the dorsum. It is closest to *E. carpenteri* (Newstead), *E. ferox* (Newstead) and possibly *E. gouligouli* Hodgson in having 6-segmented antennae, but differs from all known species in possessing a sparse submedial band of multilocular disc-pores lying between the area laterad of coxae and the submarginal band of ventral tubular ducts. In the key given in Hodgson (1991), *E. bursera* keys out to couplet 18, which should therefore be modified as shown in the accompanying key.

## Discussion

### Geographical distribution

Seventeen out of the nineteen known species of *Etiennea* occur on the African continent, the exceptions being two New World species: *E. montrichardiae* (Newstead) from Guyana, and the new species described above from Mexico, *E. bursera.* Only two non-cosmopolitan genera of Coccidae are currently believed to be restricted to Africa and South or Central America, *Alecanochiton* Hempel (including *Avricus* De Lotto, see Hodgson 1994) and *Etiennea*. Species in two other non-cosmopolitan, mainly neotropical genera: *Akermes* Cockerell and *Toumeyella* Cockerell, have been recorded from both Africa and the New World in the past. However, *Akermes colae* Green and Laing was recently placed in *Pseudocribrolecanium* Kondo (Kondo 2006), a genus currently restricted to Africa, and *Toumeyella lomagundiae* Hall and *T. obunca* De Lotto, both only known from southern Africa, have recently been transferred to a new genus (Kondo 2007). Several other genera are also known from both Africa and South or Central America: *Ceroplastes* Gray, Coccus L., *Inglisia* Maskell, *Kilifia* De Lotto, *Milviscutulus* Williams and Watson, *Parasaissetia* Takahashi, *Parthenolecanium* Sulc, *Protopulvinaria* Cockerell, *Pulvinaria* Targioni Tozzetti, and *Vinsonia* Signoret. However, as currently understood, these genera are cosmopolitan, although *Ceroplastes, Coccus, Pulvinaria* and *Saissetia* have many species that are endemic in both the Ethiopian and Neotropical regions. The disjunct distribution of these genera (plus *Etiennea* and *Alecanochiton*), suggest a Gondwanan origin, a pattern that has also been observed in many pseudococcids (Williams 1985) and eriococcids (Hoy 1962; Miller 1970; Miller and Gonsález 1975).

**Figure 1:**
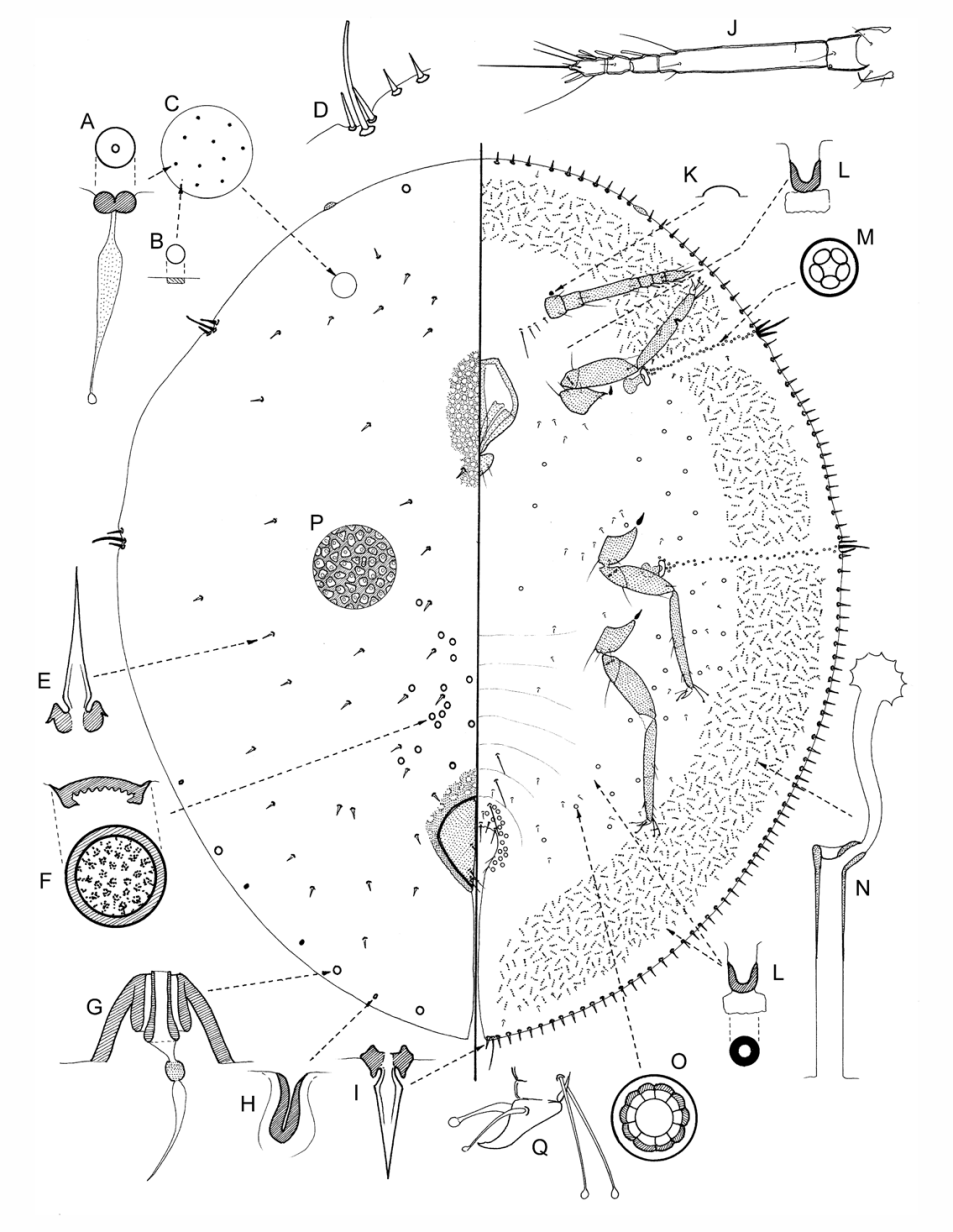
Young adult female *Etiennea bursera* n. sp., where left side of central figure represents the dorsum and right side the venter. And where A = dorsal microductule; B = simple pore; C = section of dorsum; D = stigmatic cleft; E = dorsal spinose seta; F = preopercular pore; G = dorsal tubercle; H = pocket-like sclerotization; I = marginal spinose seta; J = antenna; K = preantennal pore; L = ventral microduct; M = spiracular disc-pore; N = ventral tubular duct; O = pregenital disc-pore; P = section of dorsum of old heavily sclerotised female, and Q = claw.

### Taxonomic remarks

The above description agrees well with the generic diagnosis given in Hodgson (1991), and *E. bursera* is clearly closely related to most of the species currently included in this genus. It shares many features with *E. ferina* (De Lotto) and *E. capensis* Hodgson, i.e. presence of (i) dorsal tubercles without satellite ducts; (ii) pocket-like sclerotizations; (ii) sharply spinose marginal setae; (iii) well-developed stigmatic spines; (iv) normally developed legs; (v) multilocular disc-pores at least medio-laterally on all abdominal segments and medially on the thorax; and absence of (vi) dorsal tubular ducts, and (vii) ventral tubular ducts medially (Hodgson 1991). However, *E. bursera* differs from both *E. ferina* and *E. capensis* in having (i) only 6 antennal segments and (ii) in the absence of multilocular disc-pores medially on the venter (the other two species have 8-segmented antennae and multilocular disc-pores present medially on the venter). Two other species *of Etiennea* are known to have 6-segmented antennae, *E. gouligouli* Hodgson and *E. ferox* (Newstead). However, *E. bursera* differs from these other two species in having well-developed legs (reduced on the other 2 species). In addition *E. gouligouli* has inverted tubular ducts (absent on *E. bursera*), while *E. ferox* has very few ventral submarginal tubular ducts (much more abundant on *E. bursera*) (Hodgson 1991).

**Table:**
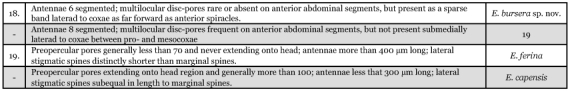
Key

Although *E. bursera* is clearly closely related to many species currently included in the genus *Etiennea*, it is not certain whether they are all congeneric with the type, *E. villiersi*. In a study of the 1^st^-instar nymphs, Kondo and Williams (2005) concluded that *E. villiersi* was close to *Hemilecanium imbricans* (Green), *H. mangiferae* Kondo and Williams and *H. theobromae* Newstead, as the crawlers of these four taxa share a range of features, particularly 2 pairs of cribriform plates. The 1st-instar nymphs of the other *Etiennea* species lack most of these characters (Hodgson 1993) - in particular the 2 pairs of cribriform plates typical of the above four species - suggesting that all but the type species might be better placed in a new genus, leaving *Etiennea* either as a monotypic genus, or as a junior synonym of *Hemilecanium*. However, no action is being taken here.

## Note

Paper copies of this article will be deposited in the following libraries. Senckenberg Library, Frankfurt Germany; National Museum of Natural History, Paris, France; Field Museum of Natural History, Chicago, Illinois USA; the University of Wisconsin, Madison, USA; the University of Arizona, Tucson, Arizona USA; Smithsonian Institution Libraries, Washington D.C. U.S.A.; The Linnean Society, London, England.
